# A Comparison of Corpectomy ACDF Hybrid Procedures with Nano‐Hydroxyapatite/Polyamide 66 Cage and Titanium Mesh Cage for Multi‐level Degenerative Cervical Myelopathy: A Stepwise Propensity Score Matching Analysis

**DOI:** 10.1111/os.13883

**Published:** 2023-09-25

**Authors:** Qiujiang Li, Bowen Hu, Umar Masood, Zhuang Zhang, Xi Yang, Limin Liu, Ganjun Feng, Huiliang Yang, Yueming Song

**Affiliations:** ^1^ Department of Orthopaedics Orthopaedic Research Institute, West China Hospital, Sichuan University Chengdu China; ^2^ Jacobs School of Medicine and Biomedical Sciences, University at Buffalo, The State University of New York Buffalo NY USA

**Keywords:** ACCF, ACDF, Adjacent segment degeneration, Cage subsidence, Degenerative cervical myelopathy, Nanohydroxyapatite/Polyamide‐66, Titanium mesh cage

## Abstract

**Objective:**

Previous studies have found satisfactory clinical results with the nano‐hydroxyapatite/polyamide 66 (n‐HA/PA66) cage to reconstruct the stability of anterior cervical column. However, studies evaluating the long‐term outcomes of the n‐HA/PA66 cage in multi‐level degenerative cervical myelopathy (MDCM) have not been reported. This study aims to compare the outcomes of corpectomy anterior cervical discectomy and fusion (ACDF) hybrid procedures between the n‐HA/PA66 cage and titanium mesh cage (TMC) to treat MDCM.

**Methods:**

After the screening for eligibility, this retrospective study involved 90 patients who underwent corpectomy ACDF hybrid (CACDFH) procedure from June 2013 to June 2018. The CACDFH procedure is the combination of ACDF and anterior cervical corpectomy and fusion (ACCF). According to the cage utilized, we categorized patients into a n‐HA/PA66 cage group and a TMC group. Then, stepwise propensity score matching (PSM) was performed to maintain comparable clinical data between groups. All the patients were followed up ≥4 years and the longest follow‐up time was 65.43 (±11.49) months. Cage subsidence, adjacent segment degeneration (ASD), segmental height (SH), segmental angle (SA), cervical lordosis (CL), and clinical data (visual analogue scale [VAS] and Japanese Orthopaedic Association [JOA] score) was evaluated preoperatively, at 1 week, and at the final surgery follow‐up. The independent student's *t* test and chi‐square test were applied to compare the differences between groups.

**Results:**

Through PSM analysis, 25 patients from the n‐HA/PA66 group were matched to 25 patients in the TMC group. The occurrence of ASD was 16.0% (4/25) in the n‐HA/PA 66 group, which was significantly less than in the TMC group at 44.0% (11/25) (*p* = 0.031). Moreover, the cage subsidence rate was significantly higher in the TMC group as compared to the n‐HA/PA 66 group (40.0% *vs.* 12.0%, *p* = 0.024). But there was no significant difference in SH, SA, and CL at any time after surgery as determined through follow‐up. The VAS and JOA scores significantly improved in both groups at 3 months postoperative and at final follow‐up. However, there were no significant differences in the VAS and JOA score at any time between the two groups in preoperative (*p* > 0.05).

**Conclusion:**

The n‐HA/PA66 cage is associated with lower rate of cage subsidence and ASD than the TMC in the treatment of MDCM. The n‐HA/PA66 cage could be superior to the TMC in corpectomy ACDF hybrid procedures.

## Graphical abstract

The findings of this study suggest that the n‐HA/PA66 cage has certain advantages over titanium mesh cage (TMC) in hybrid procedures, including a lower risk of adjacent segment degeneration and cage subsidence. Therefore, the n‐HA/PA66 cage may be more suitable than TMC in multi‐level degenerative cervical myelopathy.

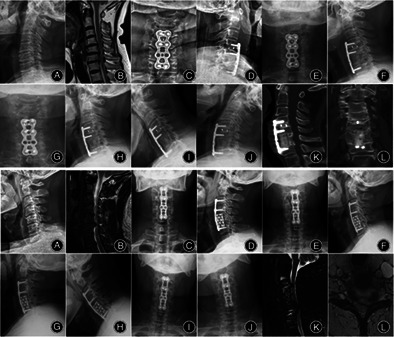

## Introduction

The available medical literature has shown that surgical procedures can improve functional status and quality of life by relieving spinal cord compression, especially in multi‐level degenerative cervical myelopathy (MDCM).^1^, ^2^, ^3^, ^4^, ^5^, ^6^, ^7^, ^8^, ^9^ Currently, anterior cervical spinal fusion surgery including anterior cervical discectomy fusion (ACDF) and anterior cervical corpectomy fusion (ACCF) are routinely used in patients with MDCM. Although ACDF can alleviate the compression by removing the disc and osteophyte directly. It maintains the structural stability of columns and restores physiological curvature with minimal blood loss, there are several risks that include inadequate decompression, limited exposure of the surgical field, and spinal cord injury.^10^ Furthermore, numerous bone‐graft interfaces increases the risk of pseudarthrosis. In contrast, ACCF has adequate surgical field exposure and complete decompression, but the damage and change to the anterior and middle columns are large, which cannot be ignored. Therefore, it may be associated with higher graft complications such as cage subsidence and displacement.^11^, ^12^ Corpectomy ACDF hybrid (CACDFH) is a combination of both ACDF and ACCF procedures. CACDFH can combine the advantages and reduce the disadvantages of ACDF or ACCF alone avoiding excessive resection of vertebras and reducing the incidence of graft‐related complications while achieving complete decompression and satisfactory clinical outcomes.^13^, ^14^

Autologous bone, allogeneic bone, and titanium mesh with autologous bone are commonly used as struts for cervical stability after CACDFH, but each of these materials has its own advantages and disadvantages.^15^, ^16^, ^17^, ^18^, ^19^, ^20^ The titanium mesh cage (TMC) is limited by its high cage subsidence rates, radiolucent opacity, and increased stress shielding effect due to the elastic modulus.^21^, ^22^, ^23^ The nano‐hydroxyapatite/polyamide‐66 (n‐HA/PA66) cage is a hollow bullet composed of a bionic composite material synthesized from nano‐hydroxyapatite and polar polymer polyamide‐66, which simulates the composition and biomechanical characteristics of natural bone.^24^, ^25^ Previous studies have found satisfactory clinical results with the n‐HA/PA66 cage to reconstruct the stability of the anterior cervical column.^26^, ^27^, ^28^, ^29^, ^30^, ^31^ However, most previous studies have primarily focused on clinical studies of one‐level ACDF or ACCF and studies evaluating the long‐term outcomes of the n‐HA/PA66 cage in MDCM have not been reported. Meanwhile, it is necessary to eliminate the effects of age, body mass index (BMI), and follow‐up time on postoperative fusion rate and cage subsidence using stepwise propensity score matching (PSM) when discussing the mid‐ and long‐term postoperative effects of the n‐HA/PA66 cage. Therefore, in this retrospective study, a confounder‐elimination process was conducted using PSM to: (i) compare the clinical and radiologic outcomes of patients with MDCM who underwent CACDFH with n‐HA/PA66 cage and TMC with at least 4 years of follow‐up; and (ii) to assess whether the chosen technique can achieve satisfactory clinical outcomes and be maintained over time during follow‐up.

## Methods

This was a retrospective, single‐center, and consecutive cohort study. We reviewed patients with three‐level MDCM who underwent CACDFH in our hospital from June 2013 to June 2018. This study was approved by the Ethics Committee of the West China hospital (No. 2019–654). Written informed consent was obtained from all participants.

### Inclusion and Exclusion Criteria

Inclusion criteria were as follows: (i) diagnosis of degenerative cervical myelopathy confirmed by imaging and physical examination；(ii) the lesions involved three levels, with ventral spinal cord compression predominating; (iii) patients underwent CACDFH in our hospital from June 2013 to June 2018; and (iv) no previous surgical intervention at cervical. Exclusion criteria were as follows: (i) patients with obvious contraindications to surgery, (ii) patients with tumors, tuberculosis, or infections; (iii) patients with serial ossification of the posterior longitudinal ligament (OPLL) and severe ossification of the ligamentum flavum; (iv) patients lacking complete clinical follow‐up data more than 4 years after surgery; and (v) patients with preoperative adjacent segmental disc degeneration (Kellgren‐Lawrence grade >3 or Pfirrmann grade >3). After exclusion and inclusion criteria were fulfilled, there were 90 patients with complete clinical data enrolled.

### Surgical Procedures

All the patients underwent routine preoperative examination, including static and lateral flexion/extension X‐ray, computed tomography (CT) scan, and magnetic resonance imaging (MRI). The surgical procedures were performed by one of four senior orthopedic surgeons with more than 20 years of experience. The level of surgery was determined by physical examination and radiological imaging. All preoperative tracheoesophageal push training was performed to prevent postoperative dysphagia. All patients received general anesthesia and a right‐sided anterior cervical approach. ACCF was performed at the severe compression levels, and the TMC or n‐HA/PA66 cage was implanted with autologous bone. Then, a one‐level ACDF was achieved on the adjacent level. Two groups patients were fixed with an Atlantis anterior cervical plate system (Medtronic Sofamor Danek, Inc., Memphis, TN, USA) to achieve immediate stabilization. All patients were required to wear a cervical collar for 12 weeks after surgery and were followed up regularly.

### Clinical Assessment

All patient‐related information was obtained from medical records. Clinical information, including age, sex, BMI, concomitant diseases (diabetes and hypertension), surgery level, follow‐up time and other general information was collected preoperatively. Clinical assessments were collected preoperatively, at 3 days postoperatively, and then intermittently until the last follow‐up (at least 4 years of follow‐up). The Japanese Orthopaedic Association (JOA) score was used to assess neurological status, and visual analogue scale (VAS) was used to assess neck pain. All patient follow‐up information was collected during outpatient visits or telephone follow‐up.

### Radiographic Measurements

**Fig. 1:**
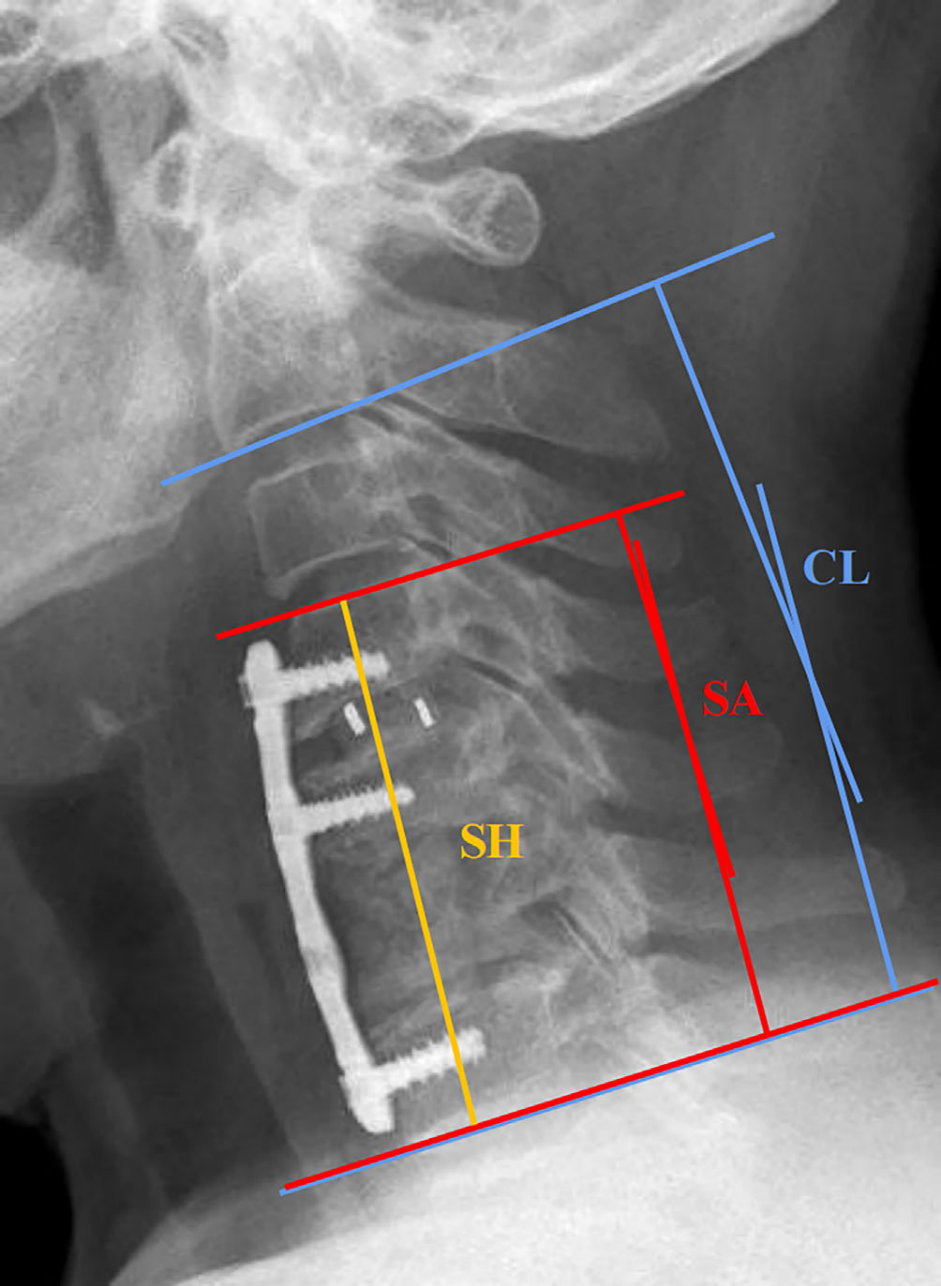
Radiological parameters measurement methods: Cervical lordosis (CL): the angle formed between the C2 and C7 lower endplate. Segmental height (SH): the distance between the midpoint of the superior endplate of the upper vertebral body and the midpoint of the inferior endplate of the lower vertebral body in the fused segment on static and lateral X‐ray. Segmental angle (SA): the angle formed between the superior endplate of the upper vertebral body and the inferior endplate of the lower vertebral body in the fused segment.

Static and lateral flexion/extension X‐ray imaging was conducted to assess the cage stability. If necessary, additional CT scan and MRI were performed. Some radiological parameters (cervical lordosis, segmental height, segmental angle) were measured preoperatively, 1 week postoperatively, and at the final follow‐up. The following terms are defined, as follows (Figure 1). Cervical lordosis (CL): the angle formed between the C2 and C7 lower endplate. Segmental height (SH): the distance between the midpoint of the superior endplate of the upper vertebral body and the midpoint of the inferior endplate of the lower vertebral body in the fused segment on static and lateral X‐ray. Segmental angle (SA): the angle formed between the superior endplate of the upper vertebral body and the inferior endplate of the lower vertebral body in the fused segment. Cage subsidence: loss of SH exceeding 3mm.^25^, ^32^ All imaging parameters were measured by two surgeons not involved in the initial procedure and quantified using the mean of the two measurements. We assigned 20 patients selected randomly for interobserver and intraobserver agreements testing. First, two spine surgeons independently measured radiographic parameters without knowledge of the clinicopathological data. Two weeks later, two spine surgeons performed a second independent measurement of the radiographic parameters. Interobserver and intraobserver agreements were evaluated based on interclass correlation coefficient (ICC) for quantitative radiographic parameters. The ICC value of the interobserver and intraobserver consistency were 0.895 and 0.891, which indicated excellent reliability. A clinical diagnosis of adjacent segment degeneration (ASD) was given when X‐ray and MRI showed one or more of the following imaging signs at adjacent segment:^33^, ^34^ (i) a decrease in the intervertebral space height greater than 3 mm on anteroposterior X‐rays; (ii) anterior or posterior displacement progression of the vertebrae greater than 3 mm on lateral X‐rays; (iii) a sagittal translation greater than 3 mm or intervertebral angle change greater than 10° on either lateral flexion/extension X‐rays; and (iv) disc degeneration progression based on Kellgren‐Lawrence classification greater than or equal to 1 grade.

### Statistical Analysis

PSM analysis was used to select and match patients from n‐HA/PA66 group to patients in the TMC group to eliminate bias in selection. Variables were selected for the PSM multivariable logistic regression model based on their effect on postoperative cage subsidence and ASD. Match tolerance of 0.2 with one‐to‐one nearest neighbor matching technique (greedy matching) without replacement was used using the “MatchIt” package in R (version 4.0.4, R Project for Statistical Computing, Vienna, Austria), as previously described by Austin.^35^ For continuous variables, normally distributed variables are expressed as mean ± standard deviation (SD), and nonnormally distributed variables are expressed as median (M) and interquartile range (IQR). Student's *t*‐test was applied to compare the differences if two group data conformed to the normal distribution, otherwise, the Wilcoxon rank‐sum test was used. Categorical data were shown as percentages and comparisons between groups were analyzed by chi‐square test or Fisher exact test. All statistical analyses were performed using SPSS 26.0 (SPSS Inc., Chicago, IL, USA) and *p*‐values <0.05 were considered statistically significant.

## Results

### Demographic Data and Clinical Characteristics before PSM

**Fig. 2:**
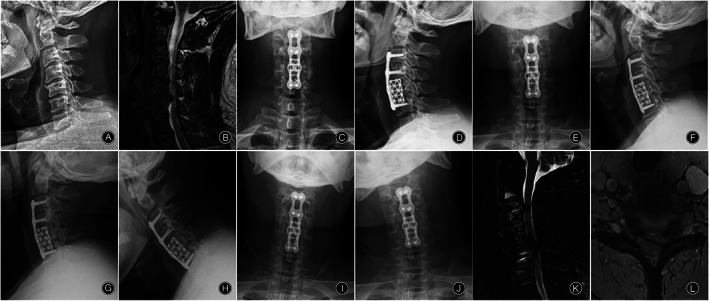
A 48‐year‐old male who underwent corpectomy ACDF hybrid procedures with a TMC for MDCM. Preoperative radiographs (A, B). 1 week X‐rays (C, D), 54 months X‐rays (E‐J) and 54 months MRI (K, L) postoperative follow‐up radiographs. 54 months after surgery, X‐rays (F‐H) showed osteophytes and narrowing of the intervertebral spaces at the adjacent segment (C6/7). Sagittal and axial MRI (K, L) at 54 months after surgery showed with disc herniation pressing on the spinal cord and nerves at the adjacent segment (C6/7).

**Fig. 3:**
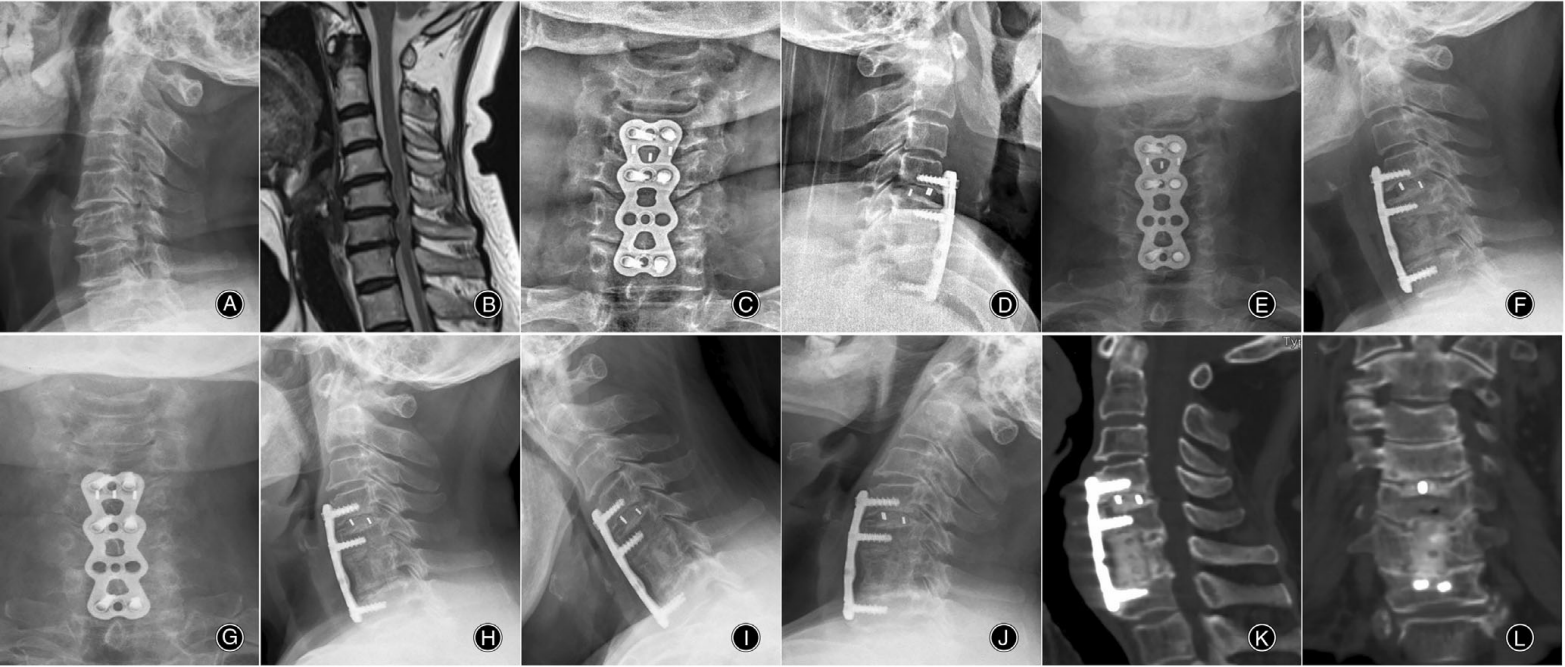
A 54‐year‐old male who underwent corpectomy ACDF hybrid procedures with n‐HA/PA66 cage for multi‐level degenerative cervical myelopathy. Preoperative radiographs (A, B). 1 week X‐rays (C, D), 1 month X‐rays (E, F), 73 months X‐rays (G‐J) and 73 months CT (K, L) postoperative follow‐up radiographs. The X‐rays (G‐J) and three‐dimensional CT scan (K, L) at 73 months after surgery showed bone fusion without internal fixation loosening, breakage, or cage subsidence.

There were 90 patients who met the criteria (38 males and 52 females; mean age 55.61 ± 9.68), including 32 in the n‐HA/PA66 group and 58 in the TMC group. Table 1 shows the comparison of demographic data and clinical characteristics data between the groups before PSM analysis. When comparing the demographic data and clinical characteristics, which included: age, sex, BMI, diagnosis, current smoker, alcohol abuse, diabetes, hypertension, and surgical intervention between the two groups, no significant differences were found except for follow‐up time. The follow‐up time was significantly longer in the n‐HA/PA66 group compared with that in the TMC group (*p* < 0.001) (see Figures 2 and 3).

**TABLE 1:** Patient demographic data and clinical characteristics between the two groups (*n* = 90)

Variables	Whole Group (*n* = 90)	n‐HA/PA66 group (*n* = 32)	TMC group (*n* = 58)	*T/X* ^*2*^ value	*p* value
Age, years	55.61 ± 9.68	57.69 ± 9.59	54.47 ± 9.62	1.524	0.132
Sex (F/M)	52/38	19/13	33/25	0.052	0.820
BMI, kg/m^2^	22.90 ± 4.83	23.01 ± 4.20	22.84 ± 5.18	1.170	0.865
Diagnosis				0.028	0.986
Radiculopathy	19 (21.1%)	7 (21.9%)	12 (20.7%)		
Myelopathy	46 (51.1%)	16 (50.0%)	30 (51.7%)		
Combined	25 (27.8%)	9 (28.1%)	16 (27.6%)		
Diabetes (yes/no)	17/73	6/26	11/47	0.001	0.980
Hypertension (yes/no)	32/58	10/22	22/36	0.402	0.526
Current smoker (yes/no)	9/81	3/29	6/52	0.022	0.883
Alcohol abuse (yes/no)	21/69	7/25	14/44	0.059	0.808
Surgery level				0.016	0.899
C3‐C6	47 (52.2%)	17 (53.1%)	30 (51.7%)		
C4‐C7	43 (47.8%)	15 (46.9%)	28 (48.3%)		
Follow‐up time, months	65.43 ± 11.49	72.03 ± 12.91	61.79 ± 8.81	0.454	<0.001

### Demographic Data and Clinical Characteristics after PSM

After PSM, there were 25 well‐balanced pairs of patients between the n‐HA/PA66 and the TMC group. The mean age for the whole cohort was 57.48 ± 8.87 years old, 32 (64.0%) were female and 18 (36.0%) were male. The average follow‐up time for 50 patients was 68.10 ± 9.11 months. After PSM, all demographic data and clinical characteristics were similar between groups (*p* > 0.05). The detailed demographic data after PSM are displayed in Table 2. Supplemental Fig. S1 described results of propensity score matching by histograms.

**TABLE 2:** Patient demographic data and clinical characteristics after propensity score matching with match tolerance of 0.2 (25 *vs.* 25)

Variable Used to match PSM	Whole Group (*n* = 50)	n‐HA/PA66 group (*n* = 25)	TMC group (*n* = 25)	*T/X* ^*2*^ value	*p* value
Age, years	57.48 ± 8.87	57.28 ± 9.40	57.68 ± 8.49	−0.158	0.875
Sex (F/M)	32/18	15/10	17/8	0.347	0.556
BMI, kg/m^2^	23.10 ± 4.82	23.18 ± 3.90	23.02 ± 5.67	0.116	0.908
Diagnosis				0.821	0.663
Radiculopathy	12 (24.0%)	5 (20.0%)	7 (28.0%)		
Myelopathy	21 (42.0%)	12 (48.0%)	9 (36.0%)		
Combined	17 (34.0%)	8 (32.0%)	9 (36.0%)		
Diabetes (yes/no)	9/41	4/21	5/20	0.136	0.713
Hypertension (yes/no)	19/31	9/16	10/15	0.085	0.771
Current smoker (yes/no)	3/47	2/23	1/24	0.355	0.552
Alcohol abuse (yes/no)	12/38	6/19	6/19	<0.001	1.000
Surgery level				0.081	0.777
C3‐C6	23 (46.0%)	11 (44.0%)	12 (48.0%)		
C4‐C7	27 (54.0%)	14 (56.0%)	13 (52.0%)		
Follow‐up time, months	68.10 ± 9.11	68.32 ± 10.23	67.88 ± 8.03	0.169	0.866

### Radiographic Outcomes

There was a mean SH increase from 7.40 ± 0.72 cm preoperatively to 7.86 ± 0.70 cm postoperatively (1 week) in the n‐HA/PA66 group. There was a mean SH increase from 7.46 ± 0.93 cm preoperatively to 7.76 ± 0.85 cm postoperatively (1 week) in the TMC group. In both groups, the mean SH slightly decreased at the final follow‐up. But, there was no significant difference in SH, SA and CL at any time after surgery. The occurrence of ASD was 16.0% (4/25) in the n‐HA/PA 66 group, which was significantly less than in the TMC group (44.0% (11/25)) (*p* = 0.031) (Table 3). Moreover, the cage subsidence rate was significantly higher in the TMC group as compared to the n‐HA/PA 66 group (40.0% *vs*. 12.0%, *p* = 0.024) (Table 3). The fusion rate at the final follow‐up was 96.0% (24/25) in the n‐HA/PA66 group and 92.0% (23/25) in the TMC group. No significant difference was observed in the fusion rate between the two groups (Table 3).

**TABLE 3:** Summary of radiographic measurements between the two groups

	n‐HA/PA66 group (*n* = 25)	TMC group (*n* = 25)	*T/X* ^*2*^ value	*p* value
SH, cm				
Preoperative	7.40 ± 0.72	7.46 ± 0.93	−0.261	0.795
Postoperative 1w	7.86 ± 0.70	7.76 ± 0.85	0.460	0.647
Final follow‐up	7.53 ± 0.68	7.29 ± 0.83	1.101	0.276
SA, °				
Preoperative	6.45 ± 5.95	5.81 ± 6.02	0.380	0.705
Postoperative 1w	14.40 ± 5.92	14.29 ± 7.19	0.063	0.950
Final follow‐up	13.03 ± 5.23	13.66 ± 6.02	−0.392	0.697
CL, °				
Preoperative	17.34 ± 9.21	18.42 ± 8.37	−0.434	0.666
Postoperative 1w	18.77 ± 9.30	19.53 ± 7.41	−0.318	0.752
Final follow‐up	19.23 ± 9.16	18.52 ± 7.12	0.303	0.763
ASD*			4.667	0.031
Yes	4 (16.0%)	11 (44.0%)		
No	21 (84.0%)	14 (56.0%)		
Cage subsidence*			5.094	0.024
Yes	3 (12.0%)	10 (40.0%)		
No	22 (88.0%)	15 (60.0%)		
Fusion rate*	24/25 (96.0%)	23/25 (92.0%)	0.355	0.552

* At final follow‐up.

### Clinical Outcomes

The VAS and JOA score significantly improved in both groups at 3 months postoperative and at final follow‐up. However, there were no significant differences in the VAS and JOA score at any time point between the two groups in preoperative evaluation (*p* > 0.05) (Table 4).

**TABLE 4:** VAS and JOA scores between the two groups

	n‐HA/PA66 group (*n* = 25)	TMC group (*n* = 25)	*T* value	*p* value
VAS arm				
Preoperative	5.28 ± 1.54	4.88 ± 1.24	1.012	0.316
Postoperative 3m	2.60 ± 0.96	2.64 ± 0.76	−0.164	0.871
Final follow‐up	2.16 ± 0.94	1.96 ± 0.74	0.836	0.407
VAS neck				
Preoperative	6.08 ± 1.50	5.48 ± 1.26	1.532	0.132
Postoperative 3m	2.52 ± 0.96	2.44 ± 0.77	0.325	0.747
Final follow‐up	2.24 ± 0.60	2.00 ± 0.71	1.297	0.201
JOA				
Preoperative	9.08 ± 3.63	10.40 ± 3.89	−1.240	0.221
Postoperative 3m	15.32 ± 3.41	16.36 ± 3.48	−1.068	0.291
Final follow‐up	15.96 ± 4.76	14.80 ± 4.32	0.903	0.371

## Discussion

Currently, several studies have confirmed that hybrid procedures can reduce surgical complications and improve the bone fusion rate.^11^, ^13^ To achieve long‐term stability, solid spinal fusion after cervical corpectomy is also very important. Previous studies have reported satisfactory clinical and imaging outcomes of one‐level ACCF with n‐HA/PA66 cage at intermediate follow‐up.^27^, ^30^ However, long‐term clinical outcomes of the n‐HA/PA66 cage in hybrid procedures have not been reported. In this study, we retrospectively analyzed 25 patients treated with hybrid procedures using n‐HA/PA66 cages and matched 25 patients with TMC. After a minimum of a 4‐year follow‐up, we found that the n‐HA/PA66 cage as a bioactive material does show a lower risk of adjacent segment degeneration and cage subsidence in hybrid procedures for MDCM, which may be more suitable than TMC in MDCM.

### Clinical Outcomes of the Two Different Cages

Preoperative neurological status is an important predictor of functional recovery after anterior cervical spine surgery. Therefore, once spinal cord compression is confirmed by a combination of clinical symptoms and imaging, early decompression should be performed. Wei *et al*.^36^ found that the ACDF combined with ACCF group had the highest JOA score and recovery rate at the final follow‐up, followed by the ACCF group and the lowest JOA score in the ACDF group. In a meta‐analysis comparing ACDF and hybrid procedures, Shamji *et al*.^37^ showed no differences in JOA, neck disability, and sagittal radiological parameters between the ACDF and Hybrid groups. In this study, we found that the JOA scores of the two groups of patients decreased after at least 4 years of follow‐up. This confirms the results reported by Yang *et al*.^30^ However, there was no significant difference in JOA scores between the two groups. The possible reasons for this may relate to the higher subsidence and lower preoperative JOA scores in patients treated with two levels of ACCF. None of the patients in our study had a severe preoperative JOA score. After fusion, the bone remodeling process is associated with cage access to the vertebral body. The study by Kim *et al*.^38^ reported that cage subsidence did not have any effect on clinical outcomes. Similarly, in our series, 13 patients with subsidence did not show poorer clinical outcomes at postoperative follow‐up. This also explains the lack of significant difference in postoperative VAS scores between the two groups of patients in our study.

### Lower Cage Subsidence in the n‐HA/PA66 Cage

There are many causes of cage subsidence, including endplate manipulation, cage size, cage position, and the material characteristics of the cage.^39^, ^40^, ^41^ In contrast, cage subsidence during follow‐up may lead to loss of intervertebral height, cervical kyphosis, foraminal stenosis, and recompression of the spinal cord and nerve roots.^42^ Hu *et al*.^27^ reported that patients treated with TMC could have a subsidence rate of 40.4%. Chen *et al*.^43^ reported TMC subsidence occurred in 19% of patients and showed that subsidence was associated with inferior clinical outcomes. The small and sharp contact surface between the ends of the TMC and the endplate may also be an important factor in the high subsidence rate of the TMC.^30^ Previous studies have reported that broadening the contact surface between the TMC and adjacent endplates can effectively reduce the incidence of subsidence.^44^ The n‐HA/PA66 cage was designed with a wider annular edge (nearly 3 mm) and greater contact surface to decrease the risk of subsidence.^25^ This also explains the lower subsidence in the n‐HA/PA66 cage group compared to the TMC group in our study (12.0% *vs*. 40.0%). In addition, a contributing factor to TMC subsidence is that it has a much higher elastic modulus than vertebral trabecular bone. The n‐HA/PA66 cage has a modulus of elasticity similar to that of natural bone, and is close to the PEEK cage in terms of biomechanics, which adequately fits the mechanical strength requirement of intervertebral fusion devices.^27^, ^30^, ^45^ After the n‐HA/PA66 cage was implanted, it could release calcium and phosphorus ions at the material‐tissue interface and provide an ideal microenvironment for osteogenesis, which may be conducive to the osteoconductive growth of bone graft and early fusion.^46^, ^47^, ^48^ This may explain the early fusion of the n‐HA/PA66 cage.

### Radiographic Outcomes of the Two Different Cages

Continued loss of intervertebral height and cervical curvature may result in secondary compression of the nerve roots and spinal cord, and subsequently increase the risk of reoperation.^49^ In the present study, a significant increase in intervertebral height occurred in both the n‐HA/PA66 cage and TMC groups after surgery and maintained stability at the last follow‐up. Recovery of cervical lordosis is an important indicator of the efficacy of anterior cervical spine surgery.^50^, ^51^ Furthermore, maintenance of cervical lordosis is a key factor in preventing deterioration of neurological function. The cervical lordosis in the TMC group remained stable as the intervertebral height decreased. Subsidence occurred mainly at the posterior border of the TMC, and the loss of height at the posterior border was much greater than at the anterior border, which could also explain the lack of significant difference in cervical lordosis between the TMC and n‐HA/PA66 cage groups. In addition, also it may also be related to the low subsidence and the small sample size of present study. Overall, These results are in accordance with the previously reported literature.^11^

### Lower Risk of ASD in the n‐HA/PA66 Cage

Adjacent segment degeneration is a common long‐term complication of laminectomy and spinal fusion procedures.^52^ The degeneration process can lead to new symptoms and the need for additional surgical interventions. Several studies have suggested that multi‐level fusion procedures are associated with a higher risk of developing ASD compared to one‐level fusion procedures.^53^ The exact mechanism behind this observation is still not fully understood, but it is believed that the added stress on the adjacent segments following a multi‐level fusion can contribute to the development of degeneration changes.^33^, ^54^ The impact of disc degeneration prior to surgery on the development of postoperative ASD also cannot be disregarded. Therefore, excluding patients with preoperative Pfirrmann grade >3 from this study can effectively reduce the effect of preoperative factors on postoperative ASD. Additionally, age, BMI, and follow‐up time have also been identified as important factors that can influence postoperative ASD. To account for these factors and ensure comparability between the study groups, PSM was performed. After PSM, our results showed that the incidence of postoperative ASD in n‐HA/PA66 group was 16.0%, which was lower than that in the TMC group, confirming previous findings. This may be due to the bioactive properties of the n‐HA/PA66 material, which has been shown to promote fusion and reduce adjacent segment degeneration. This is also the difference between our study and most of the previous studies, but also the strengths of our study. However, further studies with larger sample sizes and longer follow‐up periods are needed to fully validate these findings.

### Strengths and Limitations

Our study evaluated the long‐term outcomes of the two cages used in MDCM, and found that the n‐HA/PA66 cage had a lower risk of adjacent segment degeneration and cage subsidence than the TMC. However, there are several limitations to this study. First, this is a retrospective study that involved a single center that enrolled a relatively small number of patients. Second, selection of the cages for the procedure was not determined by chance or a randomized method, and physician‐related factors could have played a role in the results. This lack of randomization can impact the validity and generalizability of the findings, as it introduces potential biases and confounding factors. Further prospective studies with proper randomization and larger sample sizes are needed to confirm these results and draw more robust conclusions.

### Conclusions

The findings of this study suggest that the n‐HA/PA66 cage has certain advantages over TMC in hybrid procedures, including a lower risk of adjacent segment degeneration and cage subsidence. Therefore, the n‐HA/PA66 cage may be more suitable than TMC in multi‐level degenerative cervical myelopathy.

## Author Contributions

All authors had full access to the data in the study and take responsibility for the integrity of the data and the accuracy of the data analysis. Study concept and design: Q.L. and B.H. Acquisition of data: Q.L., B.H., Z.Z., and X.Y. Analysis and interpretation of the data: Q.L. and B.H. Drafting of the manuscript: Q.L., Z.Z., and B.H. Critical revision of the manuscript for important intellectual content: Umar Masood. Statistical analysis: Q.L. and Z.Z. Obtained funding: H.Y., G.F., and Y.S. Study supervision: H.Y. and Y.S.

## Conflict of Interest

The authors report no conflict of interest concerning the materials or methods used in this study or the findings specified in this paper.

## Ethics Statement

This study was performed in line with the principles of the Declaration of Helsinki. Approval was granted by the Ethics Committee of the West China hospital (No. 2019–654). Written informed consent was obtained from the parents.

## Supporting information


**Fig. S1.** Characteristic of included patients' baseline before and after propensity score matching.
