# Supplementary figures and images for: A Comparison of Corpectomy ACDF Hybrid Procedures with Nano‐Hydroxyapatite/Polyamide 66 Cage and Titanium Mesh Cage for Multi‐level Degenerative Cervical Myelopathy: A Stepwise Propensity Score Matching Analysis
Source: Orthop Surg. 2023 Sep 25;15(11):2830–8. doi: 10.1111/os.13883 (PMC10622274; doi:10.1111/os.13883)

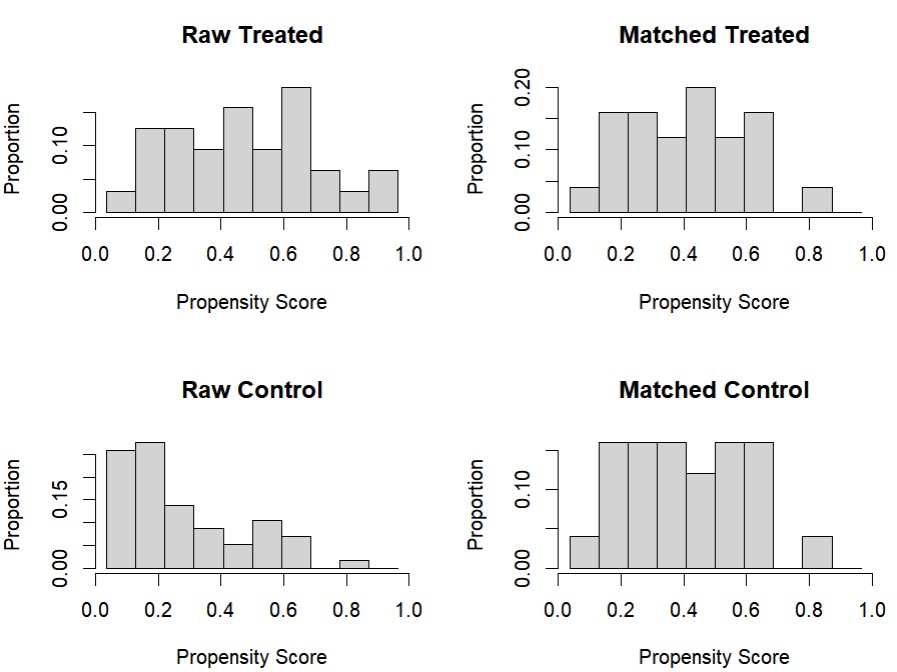

Supplement: Supplementary file 1 — Fig. S1. Characteristic of included patients' baseline before and after propensity score matching. [file OS-15-2830-s001.jpg]
